# The use of Supervised Learning to perform pairwise classification for Record Linkage over real world data

**DOI:** 10.23889/ijpds.v9i5.2902

**Published:** 2024-09-18

**Authors:** Ramon Pereira, Wagner Meira Junior, Augusto Afonso Guerra Junior

**Affiliations:** 1 UFMG

Supervised Learning (SL) is a subset of Machine Learning that uses labeled data to establish the learning process and perform tasks such as classification and regression. Record Linkage (RL) is the process for entity resolution and aims to determine if two records correspond to the same entity in the real world. A challenging aspect of RL involves estimating probabilities and weights for attributes and choosing the algorithm to perform the comparison. Our study aims to address this challenge and contribute in two primary ways: 1) by providing a labeled dataset comprising pairs of records, and 2) by evaluating an alternative classification method using an SL classifier.

We generated more than 28.000 pairs, manually reviewed, derived from a massive Record Linkage Process on the Brazilian Public Health System (SUS). From that, we selected 5000 random stratified pairs for evaluation. The data contains demographic attributes such as sex, city, date of birth, zip code, and name. We modeled the data pairwise in a vector of latent space. Our methodology involved comparing Random Forest, LightGBM, XGBoost, and Neural Networks for classification tasks on these pairs, utilizing cross-validation to optimize hyperparameters. Our findings reveal that XGBoost had the highest performance, achieving 96% accuracy.

Additionally, we conducted privacy evaluations using bloom filters, yielding an accuracy of 93%. The adoption of SL presents a promising space due to its capability for weight estimation and classification. In future endeavors, integrating these algorithms into a framework for the Record Linkage process could streamline procedures for researchers without compromising performance.

